# Reply to Harvard’s Correction

**Published:** 1904-03

**Authors:** 


### Editorial.

### REPLY TO HARVARD'S CORRECTION.

Upon another page, under the heading of "Correction from Harvard," is a communication from Dr. Eugene H. Smith, Dean of Harvard Dental School, in which he takes exception to a certain paragraph in the editorial in the February number of this journal, under the general title, "Will Dental Colleges reverse their Decision?" He especially finds subject for criticism in the third paragraph of said editorial, in which there was a mere statement of fact, as understood by the writer, in regard to what the editor pleased to call "Harvard's application."

In order that this may be understood it is necessary to quote from the paragraph in question. It is there stated, "Rumors were rife that this session [Faculties' Association at Asheville, N. C.] would witness a change of opinion and possibly an effort might be made to rescind the former action [four years' course]. The vote, however, on the *Harvard application,* refusing permission for that school to remain at three years, seemed to settle all fears in that direction, in the minds of those present, as to the stability of the four years' course."

The criticism that Dean Smith makes is, that "Harvard did not make application for permission to keep to the three years' course. Harvard simply stated her position and the reasons therefor." If this were the sole reason for the presence of the delegates from Harvard at the Asheville meeting, why, may it be asked, was it required that two representatives from that university should be sent a long journey when this could as well, and perhaps better, be stated by letter? It would seem from this acknowledgment, that the time spent at the Association of Faculties was more than wasted, and it would also seem that Harvard had decided in advance not to accept the four-year rule, no matter what might be the ultimate action of the organization. This portion of the Dean's letter admits of no other construction.

The members of the Faculties' Association did not, however, so regard it, and they considered that Harvard *did* make an application to be permitted to continue the three years' course. Both her delegates were strenuous in their statements that the entrance requirements, with a nine months' session, were in advance of other schools in membership, and should entitle Harvard to some consideration. The discussion occupied much time and the delegates from Harvard were energetic in their efforts to impress the members with the importance to the profession, and to Harvard, of these requirements. They failed, however, to make the matter clear that these were far in advance of many departments of universities connected with the Association, and possibly others. They did succeed, however, in convincing the members that Harvard proposed to advance the entrance to the Dental Department, in 1904, to the A.B. degree. Upon this understanding a committee was appointed to consider the entire question. This committee, according to Dean Smith, "failed utterly to understand the scope of our entrance requirements and reported accordingly." In view of this rather serious charge of incompetency, it may be well to state here who were appointed upon this committee. The President named Dr. J. D. Patterson, Kansas City Dental College; Dr. G. V. Black, North Western University, Chicago; Dr. H. P. Carlton, University of California; Dr. D. Stubblefield, Dental Department of Vanderbilt University, and James Truman, Chairman, University of Pennsylvania.

This committee met several times and fully considered the subject matter as stated by the delegates from Harvard. They were unanimous in the opinion that Harvard had proposed to advance the entrance requirements to the A.B. degree, and that it was expected to have this in full force by the year 1904. In view of this the committee were also decided in the opinion that this marked advance in dental education demanded peculiar consideration at the hands of the Association, and accordingly a report was prepared for presentation to the main body substantially as follows:

That in view of the fact that Harvard proposed to advance the entrance requirements, as stated, permission be granted said school to continue at three years, and further, that any other school in membership with the Association equally willing to advance to the same standard be granted a similar concession. When the Chairman presented this report, the delegates from Harvard immediately announced that they had not been understood. That

Harvard had no expectation of advancing the entrance requirements to the A.B. degree immediately, or the following year, but hoped in the course of time to reach that desirable position. That which they claimed may be best understood by quoting the remarks of Dean Smith, as officially reported in the proceedings, and which is deemed to be substantially correct.

The Dean said, "I regret that the committee has misunderstood our position in stating that the requirement is to be the A.B. degree. I have tried to make it clear that it is not our purpose, at this time, to require the A.B. degree. We state in our Catalogue that the requirements for admission will be as follows: Candidates for admission holding the degree of A.B. from any reputable college, are admitted without examination; all other candidates must qualify from the following list of studies amounting to sixteen points. (These were not given in report.) I will simply say, that, beginning in 1904, we will require the same examinations for students entering the dental school as for entering the collegiate department of Harvard College, the same in quality, but not in quantity. Now, you must bear in mind that the requirements for entrance to Harvard College differ, in this respect, from the entrance examinations of any other university in the country: that they do not accept certificates from any preparatory school. The candidate must stand an examination."

This statement of the Dean created a sensation. It was contrary to the understanding of the members of the committee, as well as that of the entire body. The report of the committee was, therefore, at once recommitted for further consideration. The committee, on reassembling, were left but one course to pursue, and, while unanimous in their decision, they reluctantly felt forced to report the following:

"Whereas, Your committee on the Harvard matter, in obedience to the direction of the Association, has reconsidered the question, and concluded that the advance proposed by the Dental Department of Harvard University, as now understood, does not warrant, at present, any reduction in the length of the course established by the Association. Therefore, be it

"*Resolved,* That the Association cannot, under the rules, consider that Harvard is entitled to less time than other schools having an equal standard of admission, with the exception, it may be, of Analytical Chemistry and Physics."

This was adopted, and at a later period in the Session the resignation of Harvard was handed in and accepted.

The assertion of the Dean, that no school in the Association demanded as much in their entrance requirements, was only true in a minor sense. Examination of the announcements of several colleges indicate very clearly that the Dean was not fully informed as to the requirements of certain schools, and, while there is a difference, it is not sufficiently marked as to place Harvard as an exceptional department. The requirements of three schools, including Harvard, each having a course of nine months, is subjoined.

REQUIREMENTS OF HARVARD DENTAL SCHOOL.

The 16 counts previously alluded to are as follows: English, 4; Physics, 2; Latin, 4; or, French, 2, and English and American History, 2; or, French, 2, and Greek and Roman History, 2; or, German, 2, and English and American History, 2; or, German, 2, and Greek and Roman History, 2. Theoretical and Descriptive (Inorganic) Chemistry and Qualitative Analysis, 4. In addition, the candidate will be obliged to offer either Algebra (2), Plane Geometry (2), or any two of the following: Solid Geometry (1), Botany (1) Zoölogy (1), Anatomy, Physiology, and Hygiene (1), Wood Working (1), Blacksmithing (1), Chipping, Filing, and Fitting (1), Machine Tool-Work (1).

The examination in English is nearly identical with that for the Department of Dentistry, University of Pennsylvania.

UNIVERSITY OF MICHIGAN, COLLEGE OF DENTAL SURGERY.

For 1903–04 the requirements are:

English. An essay of not less than five hundred words, correct in spelling, etc.

History. Myers's General History, or an equivalent, and McLaughlin's History of American Nation.

Mathematics. *Arithmetic:* Fundamental Rules, Fractions (common and decimal), Denominate Numbers, Percentage, Proportion, Involution and Evolution, and the Metric System of Weights and Measures. *Algebra:* Fundamental Rules, Fractions, Equations of the First Degree, containing two or more unknown quantities. *Geometry:* Plane Geometry. *Trigonometry:* Plane Trigonometry.

Physics. An amount represented by Avery's Natural Philosophy or Carhart and Chute's Elements of Physics.

Chemistry. General Inorganic, such as is given in Freer's or in Remsen's Elementary Chemistry.

Latin. Jones's first Latin Book, or Harkness's Latin Reader, or an equivalent amount in any other text-book, and four books of Cæsar. One year's work in German or French may be substituted for the second year of Latin.

The applicant must offer two of the following subjects: Botany, Zoölogy, Physical Geography, and Physiology.

UNIVERSITY OF PENNSYLVANIA, DEPARTMENT OF DENTISTRY, FOR 1894–95.

English. This is practically the same as Harvard.

History. (*a*) American History, with the elements of Civil Government. (*b*) General History, including Greek, Roman, and English History.

Mathematics. (*a*) *Algebra:* Fundamental operations; factors; common divisors and multiples; fractions; equations of the first degree, with one or more unknown quantities; quadratic equations; the binomial theorem. (*b*) *Plane Geometry:* as in Wentworth or Philips and Fisher.

Physics. As in Carhart and Chute or Gage's Elements.

Chemistry. As in Remsen's Elementary Course in Chemistry, or Arey's Elementary Chemistry, will be accepted in lieu of Physics.

Latin. (1) A thorough knowledge of elementary grammar. (2) Cæsar, or an equivalent course in German, French, or Spanish.

The Chemistry demanded by Harvard is part of the first-year course in this department.

A comparative examination of the requirements of these three schools give about the following in counts:

| Harvard. | | Pennsylvania. | | Michigan. | |
|---|---|---|---|---|---|
| English | 4 | English | 4 | English | 2 |
| Physics | 2 | Physics | 2 | History | 2 |
| Inorganic Chemistry | 4 | History | 2 | Physics | 2 |
| Latin | 4 | Latin | 2 | Chemistry | 2 |
| Electives | 2 | Algebra | 2 | Latin | 2 |
| | | Geometry | 2 | Science | 2 |
| | 16 | | 14 | | 12 |

These quotations certainly do not bear out the broad assertion of Dean Smith that those who have decided upon the longest term will have entered upon a "*desultory* course of four years for students insufficiently trained for a professional education." That word "desultory" has an unpleasant sound used in this connection. Our critic may have a modified meaning connected with it, but to some of us it is defined as unmethodical, unsystematic, unconnected, irregular, etc. When applied to institutions having the training of large numbers of young men, it means that the education they offer is in large degree worthless. Does Dean Smith really believe this? The writer has had a somewhat extended acquaintance with dental educational institutions for half a century, but that experience would not enable him to decide upon the effectiveness of the teaching in any school outside of the one he is regularly connected with year in and year out. He must, therefore, regard the use of the word desultory as either a slip of the pen or an unwarranted reflection on the dental colleges of the country outside of Harvard.

While it is true that the Dental Department of the University of Pennsylvania accepts diplomas of High Schools in lieu of examinations for matriculation, it must be remembered that every diploma, so presented, is passed over to the examiner appointed by the State Board of Education, and his judgment is final in regard to its value. This is not reached through a cursory examination and upon its face value, but according to the curriculum and known standing of the school. In other words, said diploma must represent a standard equal to that demanded by the University for entrance to her department. The State examiners' endorsement must, therefore, accompany every diploma before acceptance. If the applicant presents without such a diploma, the same State officer is required to make a careful examination based on the requirements for entrance to the University. The same course is pursued with all other schools in Pennsylvania, the only exception being that he follows in others the standard required by the National Association of Dental Faculties. This plan seems to the writer, to have many advantages, and avoids the charge of partiality which may be made where the examination is held in the University. It certainly has the merit that all are treated alike and that without fear or favor. The gentleman conducting these examinations is held in proper estimation not only for his general culture, but also for his wide range of information regarding the schools of the country.

It may be well to add that the reference of diplomas and examinations to this gentleman by the University of Pennsylvania is a voluntary act and made out of respect and in obedience to the laws of the National Association of Dental Faculties.

It may seem to some that the reply to the criticism of Dean Smith is given more space than is warranted, but it must be remembered that the action of Harvard, whether intended as an application for the permission of the Faculties' Association to remain at three years, or a mere statement of this school's position, has had a very injurious effect upon dental education, as a whole. This is not supposed to have been the intention, for, undoubtedly, Harvard felt that there was more than sufficient strength embodied there to stand alone, and the delegates did not, at the time, regard their action as influencing other colleges to lower their standard or that of the Faculties. It must, however, be recognized that Harvard is one of the leading dental schools of the United States, and any action taken by its governing body necessarily exercises a wide influence. The course pursued by an influential member of a community for good or ill must always be counted upon to produce a marked effect, and what is true of the individual is increasingly true of large and influential educational institutions. The moral force of the world is made strong for good or otherwise in proportion as these influences move in one direction or the other.

It is very clear to the writer that had not Harvard withdrawn, upon the plea stated, there would have been no attempt made at Buffalo, at the special meeting, to return to three years. The National Association of Dental Examiners helped along this work by their resolution passed at Asheville (INTERNATIONAL DENTAL JOURNAL, Vol. XXIV., page 905), but it is not supposed that the delegates from Harvard were aware of the action of this body, but the resolution of the Examiners as adopted was a serious blow to dental education in the United States. When a university as well sustained as Harvard should refuse to advance, it is very natural that the schools in the Association not well financially supported should be anxious to have the same privilege. It is, therefore, beyond controversy that the example of Harvard has made it very difficult to maintain the standard established by the Association of Faculties. If, however, Harvard is satisfied that the course adopted is the best for that institution, it must be left to work out the problem alone; but in doing this the officials of that department are not justified in charging lack of thoroughness upon other schools, who maintain that no undergraduate can be taught all that pertains to dentistry in three years. By dentistry is meant the collateral branches pertaining to medicine and those directly connected with its practical work. If Harvard can accomplish this in three years of nine months, congratulations are in order, but the consensus of opinion among the most advanced dental educators is, that this is impossible with the present curriculum.

